# The Sufferings of Christ

**Published:** 1887-09-17

**Authors:** 


### Words of Consolation.

[Communications for this column should be addressed, "Chaplain," care of the Editor of The Hospital, who will gratefully welcome any suggestions or inquiries from matrons, nurses, and the staff generally.]

### L.—The Sufferings of Christ.

(*For reading to the Sick.*)

Those who are most accustomed to visiting the sick and needy, seldom go into a house, or sit for long by a bedside, without hearing of some burden or tale of sadness. True sympathisers are always glad to listen. They are still more satisfied if they can take some wearied heart into their confidence, and help it to bear its load. But is it not strange, when you come to think of it in the light of reason and religion, more especially in connection with Our Lord's sufferings, that the majority of sufferers should be more troubled about others' sins than their own? or at the annoyances brought upon them by relatives or neighbours, rather than by the sting of their own misdeeds? Satan encourages this kind of dissembling. Anything to keep a sinner from feeling his own personal need of Christ. If we were clear of this deceit, we should always begin by acknowledging our own sins before we enlarged upon the shortcomings of others. Looked at by Gethsemane, little faults change their colour as well as great sins. The impure or vain thought indulged, the unkind word let slip, the petty deceit or insincerity, the harsh remonstrance, the hasty reprisal, and how many more of our failures, once regarded as so trifling, have their own burden attached, when we believe that they must have contributed to Our Lord's sorrow. Even these, if we would see sin in God's light, have to be brought separately, as it were, to the Garden of the Agony, and then on to the Cross to be crucified and killed. You can think when you have been hurt by a cut, or by a strain or bruise, the pain you suffered was quite sufficient to make you more careful under similar circumstances for the future. You may yet have to gain this kind of watchfulness in dealing with what seem trifling sins, or you will never be in earnest about avoiding them. True sorrow for them must precede their crucifixion. "Arise, let us go hence" (John xiv, 31) away from the city of false opinions, from the world's Pharisaism and disguise of sin; let us pass outside its walls down the steep bank along the stony path across the Kidron. Try to get alone with Jesus. The night is perhaps the best time. Go up the other side of that ravine so difficult to climb which leads to the naked truth about the world's sin and thine own. The path will lead thee to a garden: but no garden of the fancy, a garden of realities. There, if thou wouldst know thy burden, look beneath the long shadows of the olive-trees; look yet more earnestly than thou hast before. See Him lying so low, so bent, so humbled to the ground, so convulsed and overwhelmed. Stay by Him and watch Him. It may be thine own infirmity, so trifling in time past, may seem to be somewhat different. It may be thou wilt find cause to pray as thou never hast before, "By Thine Agony and Bloody Sweat, Good Lord, deliver me."

(*To be continued.*)

